# Generalized Survival Probability

**DOI:** 10.3390/e25020205

**Published:** 2023-01-20

**Authors:** David A. Zarate-Herrada, Lea F. Santos, E. Jonathan Torres-Herrera

**Affiliations:** 1 Instituto de Física, Benemérita Universidad Autónoma de Puebla, Apartado Postal J-48, Puebla 72570, Mexico; 2 Department of Physics, University of Connecticut, Storrs, CT 06269, USA

**Keywords:** survival probability, spectral form factor, quench dynamics, many-body quantum chaos, disordered spin model

## Abstract

Survival probability measures the probability that a system taken out of equilibrium has not yet transitioned from its initial state. Inspired by the generalized entropies used to analyze nonergodic states, we introduce a generalized version of the survival probability and discuss how it can assist in studies of the structure of eigenstates and ergodicity.

## 1. Introduction

The square overlap between a given initial state |Ψ(0)〉 and its time-evolved counterpart |Ψ(t)〉,

(1) $$SP\left(t\right)={\left|\langle \Psi \left(0\right)|\Psi \left(t\right)\rangle \right|}^{2},$$

indicates the probability of finding the system still in its initial state at time *t*. This quantity is known as survival probability, return probability, or simply the fidelity between the initial and the evolved state. This quantity has been extensively investigated since the early decades of quantum mechanics, initially in the context of the uncertainty relation between time and energy [1,2,3]. As stated by Fock in [3]:

[the time-energy uncertainty relation] may be viewed as a consequence of the general theorem of Fock and Krylov on the connection between the decay law and the energy distribution function.

The “connection” stated in the quote above refers to the fact that the survival probability (decay law) is the absolute square of the Fourier transform of the energy distribution of the initial state (energy distribution function). That is, for a state evolving according to a Hamiltonian *H*, whose eigenvalues and eigenstates are given by $E_{\alpha }$ and |α〉, one has $|\Psi \left(t\right)\rangle =\sum_{\alpha }C_{\alpha }^{\left(0\right)}e^{-iE_{\alpha }t}|\alpha \rangle$, and the survival probability can be written as:

(2) $$SP\left(t\right)={\left|\sum_{\alpha }{\left|C_{\alpha }^{\left(0\right)}\right|}^{2}e^{-iE_{\alpha }t}\right|}^{2}={\left|\int \rho \left(E\right)e^{-iEt}dE\right|}^{2},$$

where $C_{\alpha }^{\left(0\right)}=\langle \alpha |\Psi \left(0\right)\rangle$, and

(3) $$\rho \left(E\right)=\sum_{\alpha }{\left|C_{\alpha }^{\left(0\right)}\right|}^{2}\delta \left(E-E_{\alpha }\right)$$

is the energy distribution of the initial state. This distribution is also known as the local density of states (LDoS) or strength function, and its mean and variance are [4]:

(4) $$E^{\left(0\right)}=\sum_{\alpha }|C_{\alpha }^{\left(0\right)}{|}^{2}E_{\alpha }\;\mathrm{and}\;\sigma^{2}=\sum_{\alpha }{\left|C_{\alpha }^{\left(0\right)}\right|}^{2}{\left(E_{\alpha }-E^{\left(0\right)}\right)}^{2}.$$

Both the survival probability and the LDoS are studied in a variety of different fields, from quantum chaos and nuclear physics to localization and quantum information science. These quantities received significant attention from previous researchers of the Budker Institute in Novosibirsk, including those to whom we dedicated the present paper, namely Professor Giulio Casati on the occasion of his 80th birthday in 2022, Professor Felix Izrailev on the occasion of his 80th birthday in 2021, and Professor Vladimir Zelevinsky on the occasion of his 85th birthday in 2022.

Despite the simplicity of Equation (1), the evolution of the survival probability in many-body quantum systems is quite rich, with different behaviors emerging on different time scales, which reveal details about the initial state, the spectrum, and the eigenstates of the considered model. The Taylor expansion of the phase factor in Equation (2) shows that the survival probability, at very short times, t≪1/σ, presents a quadratic and universal behavior, $SP\left(t\right)\approx 1-\sigma^{2}t^{2}$, where σ is the width of the LDoS (see Equation (4)). Beyond this point, but still at short times, t≲1/σ, the decay is dictated by the shape of the LDoS. The shape of ρ(E) was investigated in [5,6] in the context of banded random matrices, while in realistic models, the transition from a Lorentzian to a Gaussian form with the increase in the perturbation strength was discussed, as in [7,8,9,10,11,12,13,14,15,16,17,18,19,20,21,22,23]. Depending on the initial state and the model considered, skewed Gaussians and bimodal distributions can also emerge [24]. Beyond the characteristic time for the initial depletion of the initial state, t∼1/σ, the survival probability exhibits a power-law decay $\propto t^{-\gamma }$ with an exponent γ that depends on the level of ergodicity of |Ψ(0)〉 and |α〉. When the LDoS is filled ergodically, γ is determined by the bounds of this energy distribution [25,26,27,28,29,30,31,32,33]. In contrast, when |Ψ(0)〉 and |α〉 are non-chaotic states, then γ depends on the level of correlations and multifractality between the states [34,35,36,37,38,39]. However, this is not yet the end of the story. In chaotic systems, where the energy-level statistics are similar to those of random matrices, the survival probability does not saturate after the algebraic decay. Instead, it reaches a value that is smaller than its infinite-time average,

(5) $$\bar{SP}=\sum_{\alpha }{\left|C_{\alpha }^{\left(0\right)}\right|}^{4},$$

and then grows in a ramp until it is finally saturated at $\bar{SP}$. The infinite time average is the last term in the equation below, which is obtained from Equation (2),

(6) $$SP\left(t\right)=\sum_{\alpha \neq \beta }|C_{\alpha }^{\left(0\right)}{|}^{2}|C_{\beta }^{\left(0\right)}{|}^{2}e^{-i(E_{\alpha }-E_{\beta })t}+\sum_{\alpha }{\left|C_{\alpha }^{\left(0\right)}\right|}^{4}.$$

The interval in which $SP\left(t\right)<\bar{SP}$ is known as the correlation hole [39,40,41,42,43,44,45,46,47,48,49,50,51,52,53,54,55,56], and there have been different methods proposed with which to measure it experimentally in systems that are out of equilibrium (see [57] and references therein). The correlation hole is a dynamic manifestation of spectral correlations and, as such, can be used to detect many-body quantum chaos in experiments that do not have direct access to the spectrum, such as experiments with cold atoms and ion traps.

In this work, motivated by generalized quantities such as Rényi entropies [58], the inverse participation ratio [59,60,61,62,63,64], and other similar quantities [65] that play a prominent role in studies of localization and multifractality, we introduce the generalized survival probability, $SP_{q}\left(t\right)$, and its corresponding generalized LDoS, $\rho_{q}^{\left(0\right)}\left(E\right)$ (see the definitions below in Equations (10) and (12), respectively). We discuss how they can help to improve our understanding of the structure of the eigenstates.

Using the one-dimensional (1D) disordered spin-1/2 model, which is often employed in the analysis of many-body localization, we compare the results for the generalized survival probability in the chaotic regime and far from it, where the duration of the power-law decay of $SP_{q}\left(t\right)$ becomes dependent on the value of *q*. We also compare the behavior of $SP_{q}\left(t\right)$ using the chaotic spin model with random matrices from the Gaussian orthogonal ensemble (GOE) and, in the latter case, provide an analytical expression for the entire evolution of the generalized survival probability.

## 2. Models

Here, we study many-body quantum systems described by the Hamiltonian:

(7) $$H=H_{0}+V,$$

where a chosen eigenstate of $H_{0}$ corresponds to the initial state, and *V* is a strong perturbation that takes the system far from equilibrium. We consider initial states that have energies $E^{\left(0\right)}=\langle \Psi \left(0\right)|H|\Psi \left(0\right)\rangle$ close to the middle of the spectrum. Two Hamiltonians *H* are investigated, of which one is a random matrix from the GOE and the other describes a 1D disordered Heisenberg spin-1/2 model.

### 2.1. Gaussian Orthogonal Ensemble

The GOE is composed of real and symmetric D×D matrices completely filled with random entries from a Gaussian distribution, with the mean zero and variance given by:

(8) $$\langle H_{jk}^{2}\rangle =\left\{\begin{array}{c} \frac{1}{2},\;\mathrm{for}\;j\neq k;\\ 1,\;\mathrm{for}\;j=k. \end{array}\right.$$

We assume that the unperturbed Hamiltonian $H_{0}$ is the diagonal part of *H* and *V* is the off-diagonal part. The model is non-physical but allows for analytical derivations that can serve as a reference for the study of realistic chaotic many-body quantum systems.

### 2.2. Disordered Spin-1/2 Model

As a physical model, we consider the 1D Heisenberg spin-1/2 model with onsite disorder, which has been used in studies of many-body localization [66,67,68,69]. The Hamiltonian is given by:

(9) $$H=\sum_{k=1}^{L}h_{k}S_{k}^{z}+J\sum_{k=1}^{L}\left(S_{k}^{x}S_{k+1}^{x}+S_{k}^{y}S_{k+1}^{y}+S_{k}^{z}S_{k+1}^{z}\right),$$

where $S^{x,y,z}$ are the spin-1/2 operators, *L* is the system size, J=1 is the coupling strength, and $h_{k}$ refers to independent and uniformly distributed random variables in [−h,h], with *h* being the onsite disorder strength. We assume periodic boundary conditions. The system conserves the total magnetization in the *z*-direction, ${\hat{S}}_{\mathrm{tot}}^{z}=\sum_{k=1}^{L}{\hat{S}}_{k}^{z}$. Throughout this paper, we work in the largest subspace, with ${\hat{S}}_{\mathrm{tot}}^{z}=0$ leading to $D=L!/\left(L/2\right){!}^{2}$. For finite sizes, *H* shows level statistics comparable to the GOE random matrices when h∼0.5, while the level repulsion fades away for h>1. We consider the unperturbed Hamiltonian to consist of the terms in the *z*-direction, $H_{0}=\sum_{k=1}^{L}\left(h_{k}S_{k}^{z}+JS_{k}^{z}S_{k+1}^{z}\right)$, and the perturbation to be the flip-flop term, $V=J\sum_{k=1}^{L}\left(S_{k}^{x}S_{k+1}^{x}+S_{k}^{y}S_{k+1}^{y}\right)$.

## 3. Generalized Survival Probability

We define the generalized survival probability as:

(10) $$SP_{q}\left(t\right)=\frac{1}{N_{q}^{2}}{\left|\sum_{\alpha =1}^{D}{\left|C_{\alpha }^{\left(0\right)}\right|}^{q}e^{-iE_{\alpha }t}\right|}^{2}={\left|\int \rho_{q}\left(E\right)e^{-iE_{\alpha }t}dE\right|}^{2},$$

where $N_{q}$ is a normalization constant given by:

(11) $$N_{q}=\sum_{\alpha =1}^{D}{\left|C_{\alpha }^{\left(0\right)}\right|}^{q},$$

where the parameter q≥0 is a positive real number, and

(12) $$\rho_{q}\left(E\right)=\frac{1}{N_{q}}\sum_{\alpha =1}^{D}{\left|C_{\alpha }^{\left(0\right)}\right|}^{q}\delta \left(E_{\alpha }-E\right)$$

is the generalized LDoS (gLDoS), with the mean and variance given, respectively, by:

(13) $$E_{q}^{\left(0\right)}=\frac{1}{N_{q}}\sum_{\alpha =1}^{D}|C_{\alpha }^{\left(0\right)}{|}^{q}E_{\alpha }\;\mathrm{and}\;\sigma_{q}^{2}=\frac{1}{N_{q}}\sum_{\alpha =1}^{D}{\left|C_{\alpha }^{\left(0\right)}\right|}^{q}{\left(E_{\alpha }-E_{q}^{\left(0\right)}\right)}^{2}.$$

The survival probability, as defined in Equation (2), and the mean and variance given in Equation (4) are recovered when q=2. For q=0, Equation (10) coincides with the spectral form factor [70], which is a quantity used to study level statistics in the time domain. Contrary to the (generalized) survival probability, the spectral form factor is not a dynamical quantity, since it does not depend on the initial state.

If one knows the generalized LDoS, we can obtain the generalized survival probability by performing the Fourier transform in Equation (10). We therefore start our analysis by examining the shape of $\rho_{q}\left(E\right)$.

### Generalized LDoS

**Figure 1:**
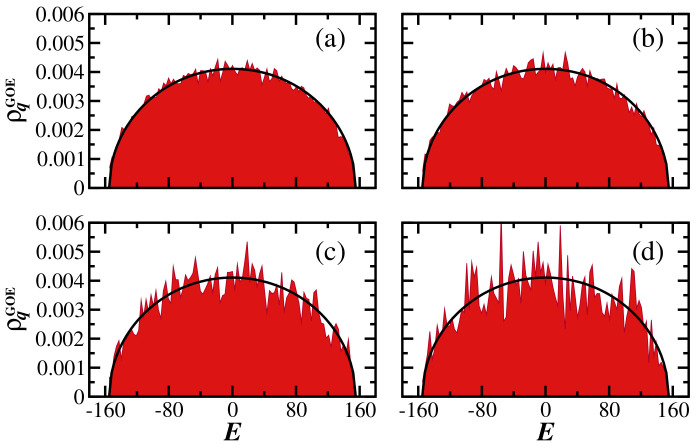
Generalized local density of states for GOE matrices for (**a**) q=0.5, (**b**) q=1.0, (**c**) q=2.0, and (**d**) q=3.0. Shaded areas are numerical results and the solid curves represent the semicircle law in Equation (14). A single disorder realization and a single initial state are considered. The matrix size is D=12,000.

Figure 1 depicts the generalized LDoS for a single random realization of a GOE matrix and different values of *q*. We observe that the semicircular shape, typical of random matrices in the limit of large D, and the length of the distribution are conserved independently of the value of *q*. This is because all eigenstates of GOE matrices are random vectors, and so is the initial state. That is, $C_{\alpha }^{\left(0\right)}$ are random numbers from a Gaussian distribution satisfying the constraint of normalization. Even though for q>1, the larger components $C_{\alpha }^{\left(0\right)}$ become enhanced, leading to the spikes observed in Figure 1c,d, the width of the distribution is not affected by *q*. This means that after averages over random realizations, one will not notice the differences between the panels. One can then state that the robustness of the generalized LDoS for different values of *q* is a sign of the ergodicity of the eigenstates of the system.

Since the components $|C_{\alpha }^{\left(0\right)}{|}^{q}$ are uncorrelated random numbers fluctuating smoothly around the average $\bar{|C_{\alpha }^{\left(0\right)}{|}^{q}}=N_{q}/D$, one can see that the gLDoS for the GOE matrices coincides with the normalized density of states $P^{\mathrm{GOE}}\left(E\right)=D^{-1}\sum_{\alpha }\delta \left(E_{\alpha }-E\right)$. Therefore, for GOE random matrices, we observe that:

(14) $$\rho_{q}^{\mathrm{GOE}}\left(E\right)=P^{\mathrm{GOE}}\left(E\right)=\frac{1}{\pi \sigma_{q}}\sqrt{1-{\left(\frac{E}{2\sigma_{q}}\right)}^{2}},$$

where the standard deviation $\sigma_{q}=\sqrt{D/2}$. In Subfigure (a) in the third figure of this same Section 3 we plot $\sigma_{q}$ as a function of *q* and confirm that $\sigma_{q}$ is, indeed, nearly constant for GOE.

**Figure 2:**
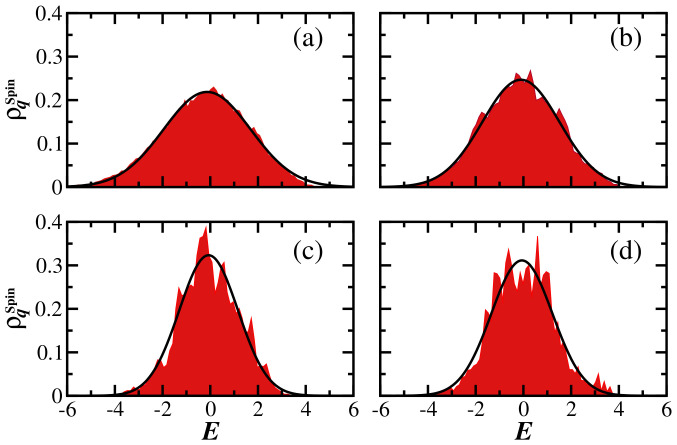
Generalized local density of states for the disordered spin-1/2 model with h=0.5 for (**a**) q=0.5, (**b**) q=1.0, (**c**) q=2.0, and (**d**) q=3.0. Shaded areas are numerical results and the solid curves represent the Gaussian expression in Equation (15). A single disorder realization is considered. The system size is L=16 with D=12,870.

For physical many-body quantum systems with two-body interactions, the density of states is Gaussian [71,72]. Thus, the expected shape of the LDoS for a system perturbed far from equilibrium and an initial state in the middle of the spectrum, as considered here, is also Gaussian, as seen in Figure 2c for q=2 and h=0.5.

Despite the persistence of the Gaussian shape for different values of *q*,

(15) $$\rho_{q}^{\mathrm{Spin}}\left(E\right)=\frac{1}{\sqrt{2\pi \sigma_{q}^{2}}}\exp\left[-\frac{{\left(E_{\alpha }-E_{q}^{\left(0\right)}\right)}^{2}}{2\sigma_{q}^{2}}\right].$$

Figure 2 makes it clear that, in contrast to the GOE, the width $\sigma_{q}$ depends on *q*. As *q* increases and the participation of the larger $|C_{\alpha }^{\left(0\right)}{|}^{q}$ becomes amplified, the width of $\rho_{q}^{\mathrm{Spin}}\left(E\right)$ becomes narrower than the density of states. This indicates that the contributions of the components at the tails of the initial-state energy distribution, where chaotic states are nonexistent, are erased.

The dependence of the width of the gLDoS on *q* reveals the limited degree of ergodicity of physical systems, even those deep in the chaotic regime. The eigenstates of physical systems are not random vectors, as in random matrices, and are not random superpositions of plane waves, as stated by Berry’s conjecture [73]. The question of how to define chaotic states in realistic systems is discussed in [10,11,74,75,76,77,78]. Our results add to these studies, providing a way to quantify the level of ergodicity in comparison to random matrices.

**Figure 3:**
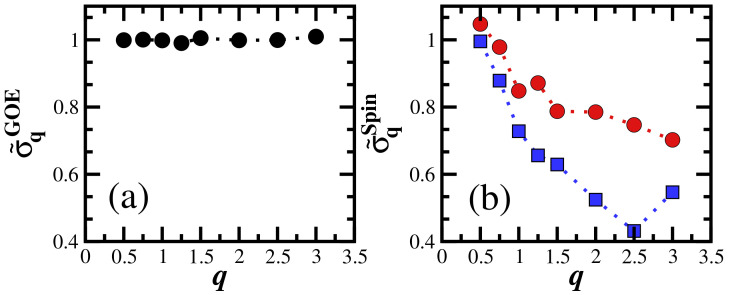
Width of the generalized LDoS normalized by the DoS for (**a**) GOE matrices, ${\tilde{\sigma }}_{q}^{\mathrm{GOE}}=\sigma_{q}^{GOE}/\sigma_{DOS}^{GOE}$, and (**b**) the spin model, ${\tilde{\sigma }}_{q}^{\mathrm{Spin}}=\sigma_{q}^{\mathrm{Spin}}/\sigma_{DOS}^{\mathrm{Spin}}$, with h=0.5 (circles) and h=2 (squares) as a function of *q*. Each point is an average over 10 disorder realizations and a single initial state. The dotted lines are guides for the eyes. D=12,000 for GOE and D=12,870 (L=16) for the spin model.

In Figure 3, we compare the results for $\sigma_{q}$ normalized by the width of the density of states (DoS) as a function of *q* for the GOE model (Figure 3a) and the spin model (Figure 3b). Each point in Figure 3 is obtained by performing an average over 10 random realizations and a single initial state. The flat curve in Figure 3a indicates the presence of fully ergodic states throughout the spectrum, while in Figure 3b, $\sigma_{q}^{Spin}/\sigma_{DOS}^{Spin}$ clearly decays as *q* increases. This occurs in the case of the chaotic model, with h=0.5 (circles), where non-chaotic states are concentrated at the edges of the spectrum, and more abruptly in the case of h=2 (squares), where non-chaotic states are also likely to be found away from the edges of the spectrum.

**Figure 4:**
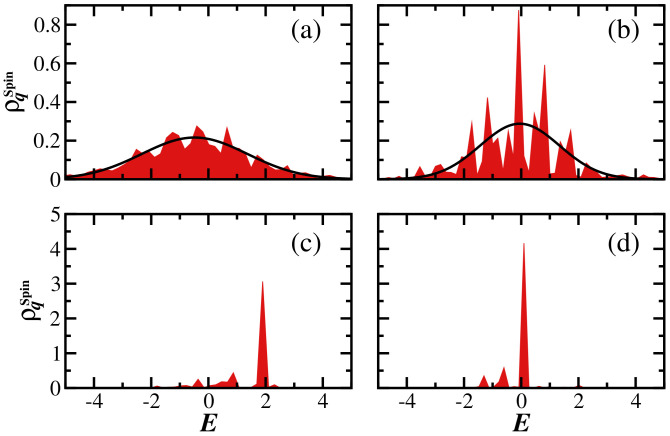
Generalized local density of states for the disordered spin-1/2 model with h=2.0 for (**a**) q=0.5, (**b**) q=1.0, (**c**) q=2.0, and (**d**) q=3.0. Shaded areas are numerical results and solid curves represent the Gaussian expression in Equation (15). A single disorder realization and a single initial state are considered. The system size is L=16 with D=12,870.

The reason for the abrupt decay of $\sigma_{q}^{Spin}$ with *q* for h=2 becomes evident in Figure 4, where we plot $\rho_{q}\left(E\right)$ for different values of *q*. When q≤1 (Figure 4a,b), the shape of the LDoS is fragmented, while for q>1 (Figure 4c,d), this structure is nearly erased, and $\rho_{q}\left(E\right)$ indicates a high degree of localization.

For finite-size systems, several numerical studies have supported the notion that the eigenstates of the disordered spin model should become multifractal in its transition to the many-body localized phase [37,64,79,80,81,82], although this has not been confirmed in the thermodynamic limit [83]. The patterns observed in Figure 4a,b also suggest fractality.

## 4. Evolution of the Generalized Survival Probability under the GOE Model: Analytical Expression

According to Equation (10), the survival probability averaged over an ensemble of initial states and random realization is written as:

(16) $$\langle SP_{q}\left(t\right)\rangle =\langle \frac{1}{N_{q}^{2}}\sum_{\alpha \neq \beta }|C_{\alpha }^{\left(0\right)}{|}^{q}{\left|C_{\beta }^{\left(0\right)}\right|}^{q}e^{-i(E_{\alpha }-E_{\beta })t}\rangle +\langle \frac{1}{N_{q}^{2}}\sum_{\alpha }{\left|C_{\alpha }^{\left(0\right)}\right|}^{2q}\rangle ,$$

where 〈⋯〉 denotes the average. The second term on the right-hand side corresponds to the infinite time average, ${\bar{SP}}_{q}$, of the generalized survival probability. For GOE random matrices, where $C_{\alpha }^{\left(0\right)}$ are random numbers from a Gaussian distribution,

(17) $${\bar{SP}}_{q}=\frac{1}{N_{q}^{2}}\sum_{\alpha }{\left|C_{\alpha }^{\left(0\right)}\right|}^{2q}=\frac{\sqrt{\pi }\Gamma \left(q+\frac{1}{2}\right)}{D\Gamma {\left(\frac{q+1}{2}\right)}^{2}}.$$

Since, for random matrices, the eigenvalues and the eigenstates are statistically independent, they can be factorized (see details in [84] and the appendix of [53]). Thus, using

(18) $$\langle e^{-i(E_{\alpha }-E_{\beta })t}\rangle =\frac{1}{D-1}\left[D\frac{J_{1}^{2}\left(2\sigma t\right)}{{\left(\sigma t\right)}^{2}}-b_{2}\left(\frac{\sigma t}{2D}\right)\right],$$

we observe that

(19) $$\frac{1}{N_{q}^{2}}\sum_{\alpha \neq \beta }|C_{\alpha }^{\left(0\right)}{|}^{q}{\left|C_{\beta }^{\left(0\right)}\right|}^{q}=1-{\bar{SP}}_{q},$$

and due to the requirement that $SP_{q}\left(t=0\right)=1$, we arrive at the analytical expression:

(20) $$\langle SP_{q}\left(t\right)\rangle =\frac{1-\langle {\bar{SP}}_{q}\rangle }{D-1}\left[D\frac{J_{1}^{2}\left(2\sigma t\right)}{{\left(\sigma t\right)}^{2}}-b_{2}\left(\frac{\sigma t}{2D}\right)\right]+\langle {\bar{SP}}_{q}\rangle .$$

**Figure 5:**
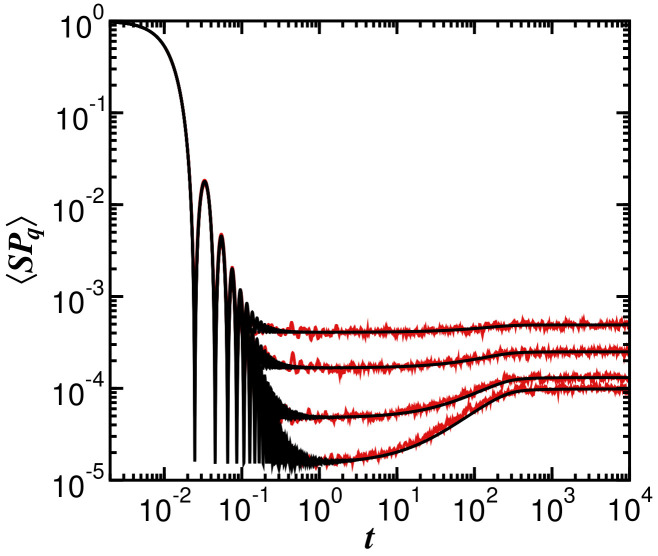
Generalized survival probability evolving under the GOE model for different values of *q*. Red curves are numerical results and the black lines correspond to the analytical expression in Equation (20). From bottom to top, q=0.5, 1.0, 2.0, and 3.0. Matrix size is D=12,000. Averages over $10^{4}$ samples.

Above, we write $\sigma_{q}=\sigma$, because $\sigma_{q}$ is constant for the GOE. The Fourier transform of the semicircular gLDoS provides the first term on the right-hand side of Equation (20), which involves the Bessel function of the first kind, $J_{1}$. This first term describes the initial decay of $\langle SP_{q}\left(t\right)\rangle$, as seen in Figure 5. It presents oscillations with the nth-zeros occurring when the initial state dynamically identifies an orthogonal state at $t_{n}∼\left(\pi n+\sqrt{2}/2\right)/2\sigma$ with n=1,2,⋯. The envelope of the oscillations decays as $t^{-3}$. The second term on the right-hand side of Equation (20), $b_{2}\left(t\right)=\left\{t\ln\left[(2t+1)/(2t-1)\right]-1\right\}\Theta \left(t-1\right)+\left[t\ln\left(2t+1\right)-2t+1\right]\Theta \left(1-t\right)$, is the so-called two-level form factor that takes $\langle SP_{q}\left(t\right)\rangle$ on a ramp to the saturation value $\langle {\bar{SP}}_{q}\rangle$.

In Figure 5, we compare the numerical results for $\langle SP_{q}\left(t\right)\rangle$ with the analytical expression in Equation (20). The agreement is excellent. The fact that $\sigma_{q}$ for the GOE model is independent of *q* (see Figure 1 and Figure 3) becomes evident, once again, in Figure 5, where the curves for the different values of *q* coincide at short times, capturing the oscillations of the Bessel function up to the minimum value of $\langle SP_{q}\left(t\right)\rangle$.

To derive the time scale, $t_{Th}^{GOE}$, where $\langle SP_{q}\left(t\right)\rangle$ reaches the minimum of the correlation hole, we must identify the point where the first and second terms in the square brackets of Equation (20) cross. Following [53], we obtain the long-term expansion of the first term in Equation (20) and expand the two-level form factor for the short times. Combining the two in the derivative of $\langle SP_{q}\left(t\right)\rangle$, we obtain:

(21) $$t_{Th}^{GOE}={\left(\frac{3}{\pi }\right)}^{1/4}\frac{\sqrt{D}}{\sigma }={\left(\frac{3}{\pi }\right)}^{1/4}.$$

To obtain the minimum value of $\langle SP_{q}\left(t\right)\rangle$ in the correlation hole, we evaluate Equation (20) at $t_{Th}^{GOE}$, which results in:

(22) $$\begin{array}{cc} {\left.\langle SP_{q}\left(t\right)\rangle \right|}_{t=t_{Th}^{GOE}} & \approx \frac{1-\langle {\bar{SP}}_{q}\rangle }{D-1}\left[\frac{D}{\pi {\left(\sigma t_{Th}^{GOE}\right)}^{3}}-\left(1-\frac{\sigma }{D}t_{Th}^{GOE}\right)\right]+\langle {\bar{SP}}_{q}\rangle \\ \; & \approx \frac{1-\langle {\bar{SP}}_{q}\rangle }{D-1}\left(-1\right)+\langle {\bar{SP}}_{q}\rangle . \end{array}$$

Finally, using Equation (17) for $\langle {\bar{SP}}_{q}\rangle$, we arrive at:

(23) $${\left.\langle SP_{q}\left(t\right)\rangle \right|}_{t=t_{Th}^{GOE}}\approx \frac{\sqrt{\pi }\Gamma \left(q+\frac{1}{2}\right)-\Gamma {\left(\frac{q+1}{2}\right)}^{2}}{D\Gamma {\left(\frac{q+1}{2}\right)}^{2}}.$$

For the particular case of q=2, Equation (23) leads to the value 2/D previously obtained in [53].

## 5. Evolution of the Generalized Survival Probability under the Spin Model

**Figure 6:**
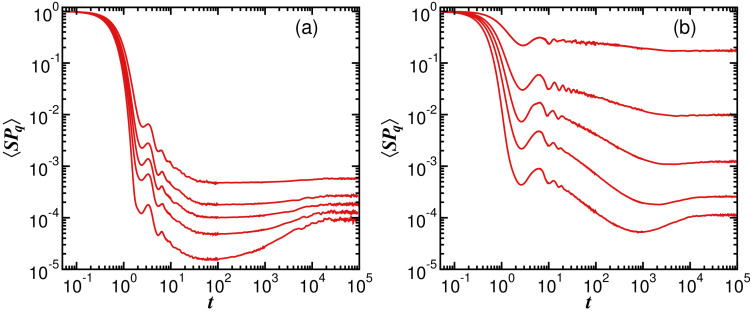
Generalized survival probability evolving under the disordered spin-1/2 model with (**a**) h=0.5 and (**b**) h=2 for different values of *q*. From bottom to top, q=0.5, 1.0, 1.5, 2.0, and 3.0. The system size is L=16. Averages over $3\times 10^{4}$ samples.

In Figure 6, we compare the evolution of $\langle SP_{q}\left(t\right)\rangle$ under the spin model for different values of *q*. In both panels, Figure 6a for h=0.5 and Figure 6b for h=2, the initial decay is determined by the envelope of the gLDoS, as seen in Equation (10). Since, according to Figure 2 and Figure 4, the shape of the distribution is Gaussian, one observes in Figure 6 that

(24) $$\langle SP_{q}\left(t\right)\rangle =\exp\left(-\sigma_{q}^{2}t^{2}\right)$$

for $t≲\sigma_{q}$. The dependence of $\sigma_{q}$ on *q* is noticeable in Figure 6a and evident in Figure 6b.

Beyond the Gaussian behavior, a power-law decay emerges:

(25) $$\langle SP_{q}\left(t\right)\rangle \propto t^{-\gamma_{q}}.$$

In Figure 6a, where the system is chaotic, the power-law exponent should depend on the bounds of the gLDoS. Since the gLDoS for the chaotic model in Figure 2 presents Gaussian tails for any *q*, we expect the same power-law exponent for all the curves in Figure 6a, which is, indeed, what the nearly parallel lines in the algebraic behavior suggest. In contrast, in Figure 6b, it is clear that $\gamma_{q}$ decreases as *q* increases, and the minimum of the correlation hole takes longer to reach. In this case, the power-law behavior reflects the correlations between the components of the initial state, which become enhanced for larger values of *q*.

## 6. Discussion

We introduced the concepts of generalized survival probability, $SP_{q}\left(t\right)$, and the generalized local density of states, $\rho_{q}\left(E\right)$. We showed that the width of the generalized local density of states, $\sigma_{q}$, depends on *q*, even when the many-body quantum system is deep in the chaotic regime, which stands in contrast with random matrices, where the width is constant and equal to the width of the density of states. Therefore, $\sigma_{q}$ may serve as a tool that can be employed to analyze and quantify the level of ergodicity of the states of physical systems with respect to random matrices.

We also showed that the power-law behavior that follows the Gaussian decay of the generalized survival probability is strongly dependent on *q* when the system is away from the chaotic regime. For a fixed value of the disorder strength, the power-law decay becomes stretched as *q* increases and the power-law exponent $\gamma_{q}$ decreases. This dependence of $\gamma_{q}$ on *q* indicates correlations between the eigenstates. In a future work, we plan to investigate how $\sigma_{q}$ and $\gamma_{q}$ may be used to study multifractality.

## Data Availability

All data are available upon request.

## Abbreviations

The following abbreviations are used in this manuscript:

DoS	Density of states
LDoS	Local density of states
gLDoS	Generalized local density of states
GOE	Gaussian orthogonal ensemble
